# Integrating transcriptomics and metabolomics for the analysis of the aroma profiles of *Saccharomyces cerevisiae* strains from diverse origins

**DOI:** 10.1186/s12864-017-3816-1

**Published:** 2017-06-08

**Authors:** Inês Mendes, Isabelle Sanchez, Ricardo Franco-Duarte, Carole Camarasa, Dorit Schuller, Sylvie Dequin, Maria João Sousa

**Affiliations:** 1 CBMA (Centre of Molecular and Environmental Biology) Department of Biology, University of Minho, Campus de Gualtar, 4710-057 Braga, Portugal; 2 INRA, UMR1083, Sciences pour l’Oenologie, Montpellier, France

**Keywords:** *Saccharomyces cerevisiae*, Wine yeast, Transcriptome, Wine flavour, Fermentation, Metabolism

## Abstract

**Background:**

During must fermentation thousands of volatile aroma compounds are formed, with higher alcohols, acetate esters and ethyl esters being the main aromatic compounds contributing to floral and fruity aromas. The action of yeast, in particular *Saccharomyces cerevisiae*, on the must components will build the architecture of the wine flavour and its fermentation bouquet. The objective of the present work was to better understand the molecular and metabolic bases of aroma production during a fermentation process. For such, comparative transcriptomic and metabolic analysis was performed at two time points (5 and 50 g/L of CO_2_ released) in fermentations conducted by four yeast strains from different origins and/or technological applications (cachaça, sake, wine, and laboratory), and multivariate factorial analyses were used to rationally identify new targets for improving aroma production.

**Results:**

Results showed that strains from cachaça, sake and wine produced higher amounts of acetate esters, ethyl esters, acids and higher alcohols, in comparison with the laboratory strain. At fermentation time T1 (5 g/L CO_2_ released), comparative transcriptomics of the three *S. cerevisiae* strains from different fermentative environments in comparison with the laboratory yeast S288c, showed an increased expression of genes related with tetracyclic and pentacyclic triterpenes metabolism, involved in sterol synthesis. Sake strain also showed upregulation of genes *ADH7* and *AAD6*, involved in the formation of higher alcohols in the Ehrlich pathway. For fermentation time point T2 (50 g/L CO_2_ released), again sake strain, but also VL1 strain, showed an increased expression of genes involved in formation of higher alcohols in the Ehrlich pathway, namely *ADH7*, *ADH6* and *AAD6*, which is in accordance with the higher levels of methionol, isobutanol, isoamyl alcohol and phenylethanol observed.

**Conclusions:**

Our approach revealed successful to integrate data from several technologies (HPLC, GC-MS, microarrays) and using different data analysis methods (PCA, MFA). The results obtained increased our knowledge on the production of wine aroma and flavour, identifying new gene in association to the formation of flavour active compounds, mainly in the production of fatty acids, and ethyl and acetate esters.

**Electronic supplementary material:**

The online version of this article (doi:10.1186/s12864-017-3816-1) contains supplementary material, which is available to authorized users.

## Background

Wine flavour is the result of the interactions between grape must components and compounds originated from microbial metabolism. Grape must is constituted by three functional groups of compounds: nutrients, flavour precursors and flavour-active non-precursors. The action of yeasts on some of these compounds, will build the architecture of the wine flavour and their fermentation bouquet. Over the past 30 years, the huge increase in the understanding of *Saccharomyces cerevisiae* metabolism*,* namely of industrial yeast strains [1] has revealed its crucial role in the development of the wine secondary aroma, with higher alcohols, acetate esters and ethyl esters being the main aromatic compounds contributing to a floral and fruity aroma [2]. Generally, wine yeast strains can be responsible for “fruity”, “floral”, “neutral”, or “cheesy”–“rancid” wine aromas, depending on their capacity to produce esters, higher alcohols, and volatile fatty acids [3]. The selection of the best wine yeast depends essentially on its oenological/phenotypic characteristics, such as fermentative rate, tolerance to ethanol and to SO_2_, response to temperature, flocculent characteristics, the presence of killer factor, malic acid metabolism and the production of several fermentation by-products, such as acetic acid, H_2_S, higher alcohols, glycerol and acetaldehyde [4–8]. A large variety of mechanisms, including heterozygosity, nucleotide and structural variations, introgressions, horizontal gene transfer and hybridization, contribute to the genetic and phenotypic diversity of *S. cerevisiae* wine yeasts [9–12], and several domestication fingerprints have been identified in their genomes [13]. Many researchers have studied the influence in the fermentation process of manipulating single genes through their deletion or over-expression, in order to clarify or to improve pathways involved in winemaking [14–17]. Some studies showed that wine strains adapt to specific oenological environments during their selection for biotechnological purposes, which is reflected in their transcriptome, proteome and metabolome [18–20]. On the other hand, transcriptome studies have been implemented using industrial yeast strains under winemaking conditions. These studies include gene expression analyses during alcoholic fermentation [20–23] and during exposure to a diversity of stresses such as high ethanol concentrations [24], low temperature [25], and high-sugar concentrations [26]. Gene expression is variable among wild-type yeast strains and it was shown that differences in gene expression during fermentation affected co-regulated genes and distinguished yeast strains [27]. Besides, winemaking strains deal better with stress-imposing environmental conditions and are able to manage nutrient deficiencies, such as nitrogen, in a more efficient and resourceful way suggesting a better adaptation to the specific stresses imposed. In order to understand the wine yeast aromatic profile, metabolomic tools are available and are commonly used. The study of metabolome includes the analysis of a wide variety of chemical compounds, usually present at very low concentrations, which is a major barrier for appropriate bioanalytical approaches. The analysis of the metabolic profile has been performed using several analytical platforms, such as gas-chromatography (GC) or liquid-chromatography (LC) coupled to mass-spectroscopy (MS) [28–30], capillary electrophoresis (CE) coupled to MS [31–34], infrared and Raman spectroscopy [35], nuclear magnetic resonance (NMR) spectroscopy [36–38] and direct injection MS (DIMS) [39, 40]. GC-MS analysis has been one of the best accepted approaches to study wine metabolome, with several advantages: sensitivity, robustness, easiness of use, low cost and ample linear range [41–44]. GC-MS combines advantages of both technologies: while MS provides individual mass spectra that can differentiate between chemically diverse metabolites, GC has high separation efficiency. The integration of the several “omic” approaches could be used to understand the variability existing within *S. cerevisiae* strains and to explore the molecular mechanisms underlying that variability.

In the present work we performed a comparative transcriptomic analysis of four *S. cerevisiae* strains from different origins and/or technological applications (wine, sake, cachaça and laboratory) at two time points during a must fermentation process and analysed the aroma profile of the fermented musts at each time point, in order to establish a correlation between gene expression and metabolite production. These strains were chosen from a larger collection as being from heterogeneous origins and displaying the biggest phenotypic differences [45], aiming to get a clearer association between flavour compounds production and gene expression.

## Methods

### Yeast strains and culture media

Four *Saccharomyces cerevisiae* strains were used in this study, in particular the commercial strain Zymaflore® VL1 (Laffort oenologie®), the cachaça strain Z63 (kindly provided by Rogélio Brandão), the sake strain Z23 (kindly provided by Gianni Liti) [46] and the laboratory strain S288c. Strains were grown at 28 °C, and routinely maintained at 4 °C on YPD plates containing 2% glucose (*w*/*v*), 2% peptone (*w*/*v*), 1% yeast extract (*w*/*v*) and 2% agar (*w*/*v*), and in glycerol (30%, *v*/v) stocks at −80 °C.

In this study, we used a natural must and a synthetic culture medium. The natural must was harvested in 2012 in the south of France (Maccabeu), flash-pasteurized and stored under sterile conditions. It contained 211 g/L of sugar and 213 mg/L of assimilable nitrogen. As a synthetic must, the MS300 (MS) medium [47] was used due to the fact that it mimics the grape musts to prepare the cells for fermentation. We inoculated 50 mL flasks containing 30 mL of YPD with cells from a Petri dish with YPD and incubated them overnight at 28 °C under stirring. Cells were then transferred to 1 L flasks containing 500 mL of MS medium in a final concentration of 2 × 10^6^ cells/mL and incubated at 28 °C with continuous stirring. The fermentation cultures in MS medium were inoculated with 2 × 10^6^ cells/mL in 1.1 L fermentors containing 900 mL of natural must.

### Must fermentations

Fermentations were performed in 1 L fermenters (NH verre) equipped with a fermentor condenser, at 20 °C, stirred continuously (100 rpm) and linked to a mass flow meter that measured the CO_2_ release rate online. CO_2_ release was determined by automatic measurements of fermentor weight every 20 min. The rate of CO_2_ production, dCO_2_/dt, is the first derivative of the amount of CO_2_ produced over time and was calculated automatically by polynomial smoothing of the CO_2_ production curve [48]. Fermentation experiments were performed in triplicate.

### Metabolite analyses

Glucose, glycerol, ethanol, pyruvate, succinic, acetic and α-ketoglutaric acids levels were analysed by high-pressure liquid chromatography (HPLC), with an Rezex ROA - Organic Acid column (Phenomenex) at 45 °C. The column was eluted with 4 mM H_2_SO_4_ at a flow rate of 0.6 mL/min. Dual detection was performed with a refractometer and a UV detector (Agilent).

Volatile aroma compounds were analyzed by GC-MS after extraction as previously described [49]. Briefly, deuterated internal standards (100 μg/L) were added to samples (5 mL) before twice extraction using 1 mL of dichloromethane. The organic phases were dried over anhydrous sodium sulphate and concentrated under nitrogen flux. Extracts were analyzed with a Hewlett Packard (Agilent Technologies, Santa Clara, California, USA) 6890 gas chromatograph coupled to a HP 5973 mass spectrometer.

### RNA isolation and sample labelling

Cells (1 × 10^9^ cells) were harvested at two time points - 5 g/L and 50 g/L of CO_2_ released - by centrifugation at 1000 g for 5 min at 4 °C and the cell pellets were washed with DEPC-treated water and then frozen in methanol at −80 °C. Total RNA was extracted with Trizol reagent (Gibco BRL, Life Technologies) and was purified with the RNeasy kit (Qiagen). The quantity and the quality of the extracted RNA were checked by spectrometry (NanoDrop 1000, Thermo Scientific). We used the Agilent 8x15k gene expression microarrays (Design ID 016322, Agilent Technologies, Santa Clara, CA, USA) according to the manufacturer’s instructions. Fluorescent cRNAs were synthesized from 100 ng of total RNA using the One color RNA Spike-In kit (Agilent Technologies). Labeled cRNA was purified with the RNeasy Kit (Qiagen). Microarrays were hybridized for 17 h at 65 °C in a rotating hybridization oven (Corning), with the Gene Expression Hybridization kit (Agilent). The hybridization signal was detected with a GenePix 4000B laser scanner (Axon Instruments).

### Statistical analysis

Statistical analyses were performed using R software, version 3.0.3 [50]. To obtain a general overview of the production of volatile compounds during the fermentation for each stage of fermentation (T1 and T2), principal component analysis (PCA) was performed using the FactoMineR package [51].

The limma package [52] was used to import and normalize the global microarray data (quantile method for normalization between arrays). For each studied time of CO_2_ released (T1 and T2) and based on this normalized dataset of 6200 points for the 4 strains, we used a sparse partial least square – discriminant analysis (sPLS-DA), an exploratory approach in a supervised context in order to select the most important transcripts relative to the 4 strains [53]. We tuned the number of dimensions of the sPLS-DA to 2 and the number of variables to choose on these 2 dimensions to 400.

A functional analysis was performed on the selected transcripts by time point, in order to highlight significant functional groups according to the Gene Ontology (GO) process terms using the GeneCodis program with the FDR method at a *p* value cutoff of 0.05 [54].

For each time point, a multivariate factorial analysis (MFA) was also performed to obtain an overview of the dataset, which consisted in 433 variables measured for 4 strains (S288c, VL1, cachaça, sake). The data set included a group of individuals described by two types of variables: the normalized expression of the 400 transcripts selected by the sPLA-DA according to the 4 strains, and the 33 volatile compounds produced during the fermentation by the 4 strains. The MFA takes into account the structure of the two groups of data and balances the influence of each group of variables. This enables the study of links between expression data and volatile compounds production [51].

Microarray data accession numbers: the complete data set is available through the Gene Expression Omnibus (GEO) database. The microarray description is under GEO accession number GPL16244.

## Results and discussion

### Fermentative profiles and metabolic characterization

**Fig. 1:**
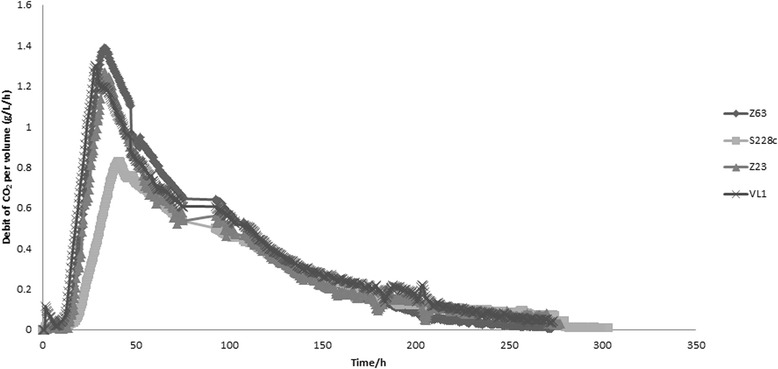
Fermentation profiles of the four strains used in this study in respect to debit of CO_2_ per volume (g/L/h) per time (h-hours). Values are the averages from 3 biological replicates. Fermentations were carried out at 20 °C (100 rpm) using Maccabeu grape must

Aiming at a better understanding of the molecular and metabolic bases of aroma production during a fermentation process, we started by characterizing fermentative profiles and metabolite production of grape must fermentations conducted by three *Saccharomyces cerevisiae* strains isolated from different fermentative environments, namely cachaça Z63, sake Z23 and the commercial wine yeast VL1, as well by the laboratory reference strain S288c. These strains were previously characterized genetically and phenotypically [45, 55] and were selected from a larger yeast collection based on their dissimilarities [45]. Triplicate fermentations were carried out with each of the four strains using natural must Maccabeu. The fermentation performance of the strains is presented in Fig. 1, in which each curve represents the average debit of CO_2_ from the three replicates for each strain. With the exception of the laboratory strain, for which a slower fermentation and a lower maximum fermentation rate were obtained, the remaining three strains present a similar fermentative profile with a V_max_ between 1.2 and 1.4 g/L/h of CO_2_ released.

In order to obtain a characterization of their metabolic profile, high-performance liquid chromatography (HPLC) and gas chromatography – mass spectrometry (GC-MS) analysis were performed with samples from two time points of fermentation: exponential phase (T1, 5 g/L of CO_2_ released) and stationary phase (T2, 50 g/L CO_2_ released). Thirty-eight compounds were quantified including 11 ethyl esters, 7 acetate esters, 4 organic acids, 5 higher alcohols, 10 volatile fatty acids and propanol (Additional file 1: Table S1).

**Fig. 2:**
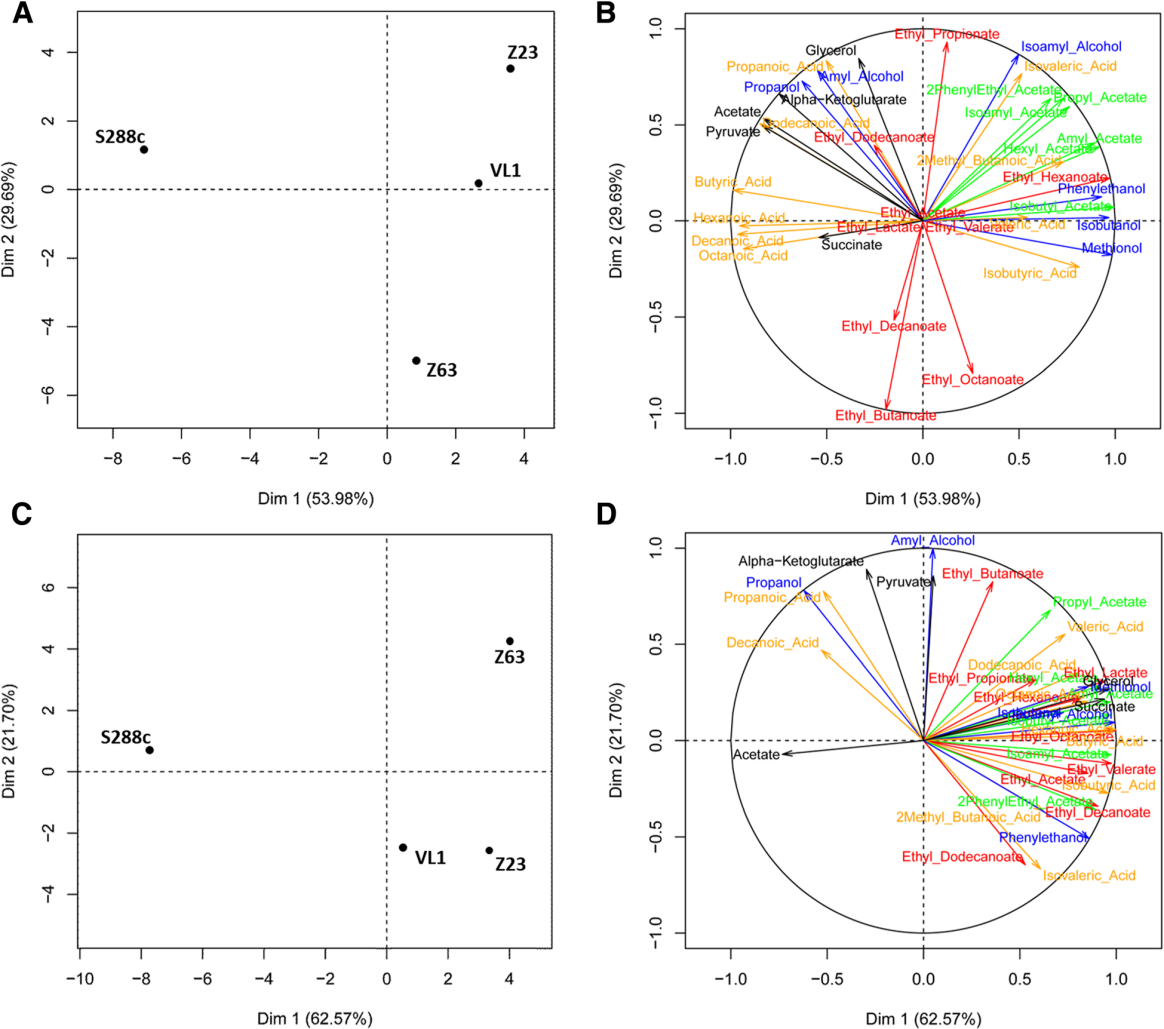
Principal component analysis of GC-MS and HPLC data for the four strains tested: **a** – four *S. cerevisiae* strains (scores) analysed by GC-MS and HPLC at T1 (5 g/L). **b** – concentration of volatile compounds detected by HPLC and GC-MS at T1 (5 g/L). **c** – four *S. cerevisiae* strains (scores) analysed by GC-MS and HPLC at T2 (50 g/L). **d** – concentration of volatile compounds detected by HPLC and GC-MS at T2 (50 g/L)

PCA analysis based on the compounds quantified both by HPLC and GC-MS (Fig. 2) showed intra-strain differences, with a discrimination of the laboratory strain from the other three strains at T1 (Fig. 2a) and T2 (Fig. 2c). Circles of correlation (Figs. 2b, d) show the contribution of each quantified metabolic compound to the separation of the strains in the scores plot. Only the first two components were considered, since they explain a high percentage of the variability found between isolates and between compounds: 83.7% and 84.3% for T1 and T2, respectively. At T1 (Figs. 2a and b), a clear differentiation between laboratory strain and the other three strains was obtained according to the first axis. Productions of acetate esters (green) and of some higher alcohols (blue) had positive contributions to this axis while formation of medium chain fatty acids (hexanoic, octanoic and decanoic acids) was negatively involved. Strain Z63, having its origin in the fermentative beverage cachaça, distinguished along the second axis by a higher production of ethyl decanoate, ethyl octanoate and ethyl butanoate, compared with other tested strains.

At time-point T2, corresponding to the stationary phase of fermentation, a similar scenario was observed, with a clear separation of laboratory strain S288c from the others according to the first axis, and a separation of strain Z6 3 (cachaça) from strains Z23 and VL1 along the second one. However, the major contributors to the two axes differed between the two time points. During the stationary phase, fermentation by strains Z63, Z23 and VL1 produced higher amounts of almost all metabolites assessed, in comparison with the laboratory strain: acetate esters, ethyl esters, the majority of the acids apart from decanoic and propanoic acids and most of higher alcohols except propanol (first axis). From the three ethyl esters produced highly by cachaça strain at T1, only ethyl butanoate was again responsible for the separation of this strain from strains VL1 and Z23 (second axis).

Our results show that at the two time points considered in this work, the compounds contributing the most to the strains separation in comparison with S288c were the acetate and ethyl esters and the higher alcohols. It is well known that higher alcohols have positive effect on wine aroma as well [3, 56]. In the same way esters, produced by yeasts during alcoholic fermentation, have a significant influence on the fruity aromas of the final product, both in the case of ethyl fatty acid esters and acetate esters [57, 58]. So the results indicate that must fermentations carried with yeasts isolated from any of the three wild fermentative environments will be characterized by a higher development of the “yeast bouquet” and originate wines with much more complex aroma and flavour, than the laboratory strain used as reference. In addition, the aroma profile of sake strain will be closer to the one of the wine strain. In the case of volatile fatty acids, their concentration varied from 82 to 220 mg/L at T1 and 81 to 289 mg/L at T2, influencing also the PCA position of the analysed strains. The concentration of volatile acids is of particular relevance since in concentrations above 300 mg/L they are associated with unpleasant odors and tastes, such as a pungent smell and taste. In concentrations below that level, volatile acids can have a positive impact with fruity and floral aromas [59], mainly due to the inhibition of their esters hydrolysis.

### Comparative transcriptomics

Comparative transcriptomics of the three *S. cerevisiae* strains isolated from the different fermentative environments in comparison with the reference yeast S288c was conducted using Agilent 8x15k microarrays. mRNA samples were collected at the two time points T1 and T2, as explained in the previous section.

Tables 1, 2, 3 and 4 summarize the main findings obtained with transcriptomic characterization of the three fermentation isolates, in comparison with laboratory strain S288c. Results were analysed using Funspec with Bonferroni correction (*p* < 0.05), and down or upregulated genes are indicated for the three strains in comparison with S288c, both at T1 (Tables 1, 2) and T2 (Tables 3, 4). Genes were categorized in accordance with MIPS Functional Catalogue [60], and the ones common to the three strains are underlined.

**Table 1:** Categorization of genes with significantly decreased expression (Bonferroni *p* < 0.05) in Z63, Z23 and VL1 strains in comparison to S288c, at T1 (5 g/L of CO_2_ released). Genes common to the three strains are underlined

MIPS functional category	Strain		
	Z63	Z23	VL1
pheromone response, mating-type determination, sex-specific proteins	AFR1 ASG7 BAR1 DIG1 EXG1 FAR1 FUS1 FUS3 GIC2 GPA1 HO MFA1 MFA2 PHO81 PRY1 RDH54 SPA2 SST2 STE18 STE2 STE23 STE4 STE5 STE6 UBC4	AGA1 ASG7 BAR1 DIG1 FAR1 FUS1 FUS3 GIC2 GPA1 HO HSP82 MFA1 MFA2 PHO81 PRY1 RDH54 SST2 STE18 STE2 STE23 STE4 STE5 STE6	AFR1 ASG7 ASH1 BAR1 BEM1 CLN2 DIG1 FAR1 FUS1 FUS3 GFA1 GIC2 GPA1 HO MCK1 MFA1 MFA2 PHO81 PRY1 RDH54 SAG1 SAN1 SIR2 SST2 STE18 STE2 STE23 STE4 STE5 STE6
degradation of asparagine, metabolism of aspartate	ASP3–1 ASP3–2 ASP3–3 ASP3–4	ASP1 ASP3–1 ASP3–2 ASP3–3 ASP3–4	ASP1 ASP3–1 ASP3–2 ASP3–3 ASP3–4

**Table 2:** Categorization of genes with significantly increased expression (Bonferroni *p* < 0.05) in Z63, Z23 and VL1 strains in comparison to S288c, at T1 (5 g/L of CO_2_ released). Genes common to the three strains are underlined

MIPS functional category	Strain		
	Z63	Z23	VL1
electron transport and membrane-associated energy conservation	ATP20 COR1 COX1 COX13 COX3 COX5A COX6 COX7 CYB2 CYC1 CYC7 MCR1 NDE1 NDI1 QCR10 QCR2 QCR6 QCR7 QCR8 QCR9 RIP1	COB COX1 COX3 COX5A CYB2 CYC1 CYC7 NDE1 NDI1 QCR8 QCR9 RIP1	ATP20 COX1 COX13 COX5A COX6 COX7 CYB2 CYC1 CYC7 NDI1 QCR10 QCR7 QCR8 QCR9 RIP1
aerobic respiration	COR1 COX1 COX13 COX23 COX3 COX5A COX6 COX7 CYT1 ISF1 MAM33 MBR1 NDE1 NDI1 PET10 PET9 QCR10 QCR2 QCR6 QCR7 QCR8 QCR9 RIP1	COB COX1 COX16 COX23 COX3 COX5A CYT1 MAM33 NDE1 NDI1 QCR8 QCR9 RIP1 YDR115W	COX1 COX13 COX16 COX23 COX5A COX6 COX7 CYT1 MRPL1 NDI1 QCR10 QCR7 QCR8 QCR9 RIP1 YDR115W
tetracyclic and pentacyclic triterpenes (cholesterin, steroids and hopanoids) metabolism	ARE2 ERG1 ERG10 ERG13 ERG2 ERG27 ERG28 ERG5 ERG7 ERG9 HMG1 IDI1 MCR1 MVD1 NSG2 OSH6	ARE2 ERG1 ERG13 ERG2 ERG28 ERG5 ERG9 HMG1 IDI1 MVD1 NSG2 OSH6	ARE2 ERG1 ERG2 ERG27 ERG28 ERG5 ERG9 HMG1 IDI1 MVD1 NSG2
mitochondrion	CYB2 HSP10 MBR1 MDM35 MDV1 MNP1 MRM2 MRP2 MRP21 MRPL13 MRPL20 MRPL23 MRPL32 MRPL35 MRPL37 MRPL38 MRPL39 MRPL40 MRPL44 MRPL6 MRPL9 MRPS28 NDE1 NDI1 RSM25 TIM10 YMR31	CYB2 HSP10 MNP1 MRP13 MRP2 MRP21 MRPL10 MRPL13 MRPL19 MRPL20 MRPL23 MRPL27 MRPL32 MRPL35 MRPL37 MRPL38 MRPL39 MRPL4 MRPL40 MRPL44 MRPL6 MRPL8 MRPL9 MRPS16 MRPS28 NAM9 NDE1 NDI1 RML2 RSM18 RSM19 RSM25 RSM26 TIM10 YDR115W YMR31	CYB2 GET1 HSP10 MDM35 MDV1 MNP1 MRM2 MRP13 MRP2 MRP21 MRP49 MRPL1 MRPL10 MRPL13 MRPL20 MRPL23 MRPL27 MRPL32 MRPL35 MRPL36 MRPL37 MRPL38 MRPL39 MRPL40 MRPL44 MRPL49 MRPL6 MRPL9 MRPS16 MRPS28 NDI1 PET18 RSM18 RSM19 RSM25 RSM26 SAM37 TIM10 TIM12 TIM9 YDR115W YMR31
ribosomal proteins		MNP1 MRP13 MRP2 MRP21 MRPL10 MRPL13 MRPL19 MRPL20 MRPL23 MRPL27 MRPL32 MRPL35 MRPL37 MRPL38 MRPL39 MRPL4 MRPL40 MRPL44 MRPL6 MRPL8 MRPL9 MRPS16 MRPS28 NAM9 RML2 RPL19A RPL22A RPL34A RPL36A RPS10B RPS14B RPS17A RPS21B RPS24A RPS27A RSM18 RSM19 RSM25 RSM26 YDR115W YMR31	MNP1 MRP13 MRP2 MRP21 MRP49 MRPL1 MRPL10 MRPL13 MRPL20 MRPL23 MRPL27 MRPL32 MRPL35 MRPL36 MRPL37 MRPL38 MRPL39 MRPL40 MRPL44 MRPL49 MRPL6 MRPL9 MRPS16 MRPS28 RPL11A RPL11B RPL18B RPL19A RPL19B RPL20B RPL22A RPL23A RPL26A RPL27A RPL30 RPL33B RPL34A RPL35B RPL36A RPL38 RPL40A RPL43B RPL9B RPP1A RPS10A RPS10B RPS14B RPS16A RPS17A RPS18B RPS21A RPS21B RPS24A RPS24B RPS25A RPS26A RPS27A RPS30A RPS30B RPS6B RPS8B RPS9A RSM18 RSM19 RSM25 RSM26 SWS2 YDR115W YMR31
Fermentation		AAD16 AAD4 AAD6 ADH7 ALD2 MSC7 YPL088W

**Table 3:** Categorization of genes with significantly decreased expression (Bonferroni *p* < 0.05) in Z63, Z23 and VL1 strains in comparison to S288c, at T2 (50 g/L of CO_2_ released)

MIPS functional category	Strain		
	Z63	Z23	VL1
degradation of asparagine, metabolism of aspartate	-	ASP1 ASP3–1 ASP3–2 ASP3–3 ASP3–4	ASP1 ASP3–1 ASP3–2 ASP3–3 ASP3–4
ribosomal proteins	-	MDN1 PIH1 RPL11A RPL11B RPL12A RPL13A RPL15A RPL16A RPL16B RPL22A RPL22B RPL23A RPL30 RPL32 RPL33B RPL34A RPL43A RPL8A RPS0B RPS11A RPS13 RPS18B RPS1B RPS24A RPS24B RPS27A RPS29B RPS4A RPS5 RPS6A

**Table 4:** Categorization of genes with significantly increased expression (Bonferroni *p* < 0.05) in Z63, Z23 and VL1 strains in comparison to S288c, at T2 (50 g/L of CO_2_ released)

MIPS functional category	Strain		
	Z63	Z23	VL1
electron transport and membrane-associated energy conservation	-	-	ATP20 COR1 COX1 COX5A COX6 COX7 CYB2 CYC1 CYC7 NDI1 PMA2 QCR2 QCR7 RIP1
tetracyclic and pentacyclic triterpenes (cholesterin, steroids and hopanoids) metabolism	ARE2 ERG1 ERG10 ERG13 ERG2 ERG20 ERG24 ERG27 ERG28 ERG5 ERG6 ERG9 HMG1 IDI1 MVD1 NCP1	-	ARE2 ERG1 ERG10 ERG12 ERG13 ERG2 ERG20 ERG24 ERG25 ERG26 ERG27 ERG28 ERG5 ERG6 ERG7 ERG9 HMG1 IDI1 MVD1 NCP1
Mitochondrion	CLU1 HOT13 HSP10 MDH1 MDM35 MRP2 MRP49 MRPL11 MRPL13 MRPL20 MRPL23 MRPL27 MRPL32 MRPL35 MRPL38 MRPL4 MRPL6 MRPL8 MRPS28 NDI1 PET18 PNT1	-	-
fermentation	-	AAD15 AAD3 AAD4 AAD6 ADH7 ALD2 ALD6	AAD15 AAD3 AAD4 AAD6 ADH6 ADH7 ALD6 MSC7

As to time point 1 (T1), analysis of Table 1 shows that one group of genes related with the functions “pheromone response, mating-type determination, sex-specific proteins”, was downregulated in all three strains. Since the 3 isolates used in the present work are diploid [46, 55, 61], and the laboratory strain S288c used for comparison is haploid [62], differences in ploidy could thus underlie the differences in expression of the genes related with the mating and the pheromone response. Genes involved in the degradation of asparagine/metabolism of aspartate (*ASP3–1*, *ASP3–2*, *ASP3–3* and *ASP3–4*) appeared as downregulated in the three isolates, and *ASP1* coding for cytosolic L-asparaginase was downregulated in Z23 and VL1 strains. This is likely related with the fact that some *S. cerevisiae* strains, including some wine and sake strains, had lost the *ASP3* locus [63].

Genes with significantly increased expression at T1, include a group of genes related with tetracyclic and pentacyclic triterpenes metabolism (cholesterin, steroids and hopanoids) that was upregulated in the 3 strains comparatively to the laboratory strain (Table 2). Most of these genes are involved in sterol synthesis namely ergosterol, which by contributing to the fluidity of the yeast membrane, allows a more efficient activity of membrane transporters and increased tolerance to ethanol [64], correlating with the superior fermentation performances of strains. The higher sterol biosynthesis could also divert acetyl CoA from fatty acid biosynthesis, so the lower levels of these genes in S288c strain could explain the higher production of medium chain fatty acids (MCFA) by this strain (Fig 2b). Several genes involved in aerobic respiration, electron transport and mitochondrion were also upregulated in the three mentioned strains in comparison with S288c (Table 2), suggesting a less strict glucose repression in the strains isolated from the fermentative environments. The higher respiratory capacity might also be associated with the higher production of fusel acids (Fig. 2), due to lower need to reoxidize NADH through the Ehrlich pathway [3]. Also, at T1, the increased expression in Z23 of genes related with aldehyde oxidation, namely *AAD4, AAD6, AAD16* and *ADH7,* might relate with the higher production of fusel alcohols in this strain especially of isoamylalcohol, phenylethanol, isobutanol and methionol (marked in blue in Fig. 2b).

Regarding time point T2 (Table 3), there were no common downregulated genes in the three characterized strains. Genes related with ribosomal proteins were downregulated only in sake strain (Table 3). The differences in the expression of these genes, observed also at T1 for Z23 and VL1 strains, may originate from the different fermentative profile and the different metabolic stage of each strain, at this time point. Regarding upregulated genes (Table 4), a group of genes involved in the synthesis of sterols was still upregulated for the cachaça (Z63) and wine (VL1) strains. For the sake strain (Z23) these genes were similarly expressed when compared to the laboratory strain suggesting that sake strain could be in an less active metabolic stage, in comparison with the other strains, requiring less sterol synthesis, which is also in agreement with the observed repression of ribosomal genes. Also at T2 it is visible that some genes upregulated in strains Z23 and VL1 (*ADH7*, *ADH6* and *AAD6)* are involved in the Ehrlich pathway and so related with the formation of specific compounds, such as higher alcohols. In accordance with these results, metabolic analysis showed an increase of the same higher alcohols for T2 in comparison with T1, namely: methionol, isobutanol, isoamyl alcohol and phenylethanol. The only alcohols that seem not to be included in this association are amylalcohol and propanol, which were equal or less produced, respectively, in these strains in relation to S288c. The differential production of acetate esters by the two groups of strains (marked in orange in Figs. 2b and d) could be related with the differences in expression of *ALD6* [65], which was overexpressed in strains Z23 and VL1. This gene is involved in the formation of acetic acid that can then be converted into acetyl-CoA and subsequently incorporated in acetate esters.

Similarly to the downregulated genes, at T2 there were no common upregulated genes for the three strains. This is opposite to the observed at T1 and may reflect that the differentiation of the strains, isolated from different fermentation processes, is especially important enduring the multistress stationary phase of fermentation where each strain developed different adaptive mechanisms in response to the specific fermentation conditions [46].

### Combined transcriptomics and metabolomics analysis

**Fig. 3:**
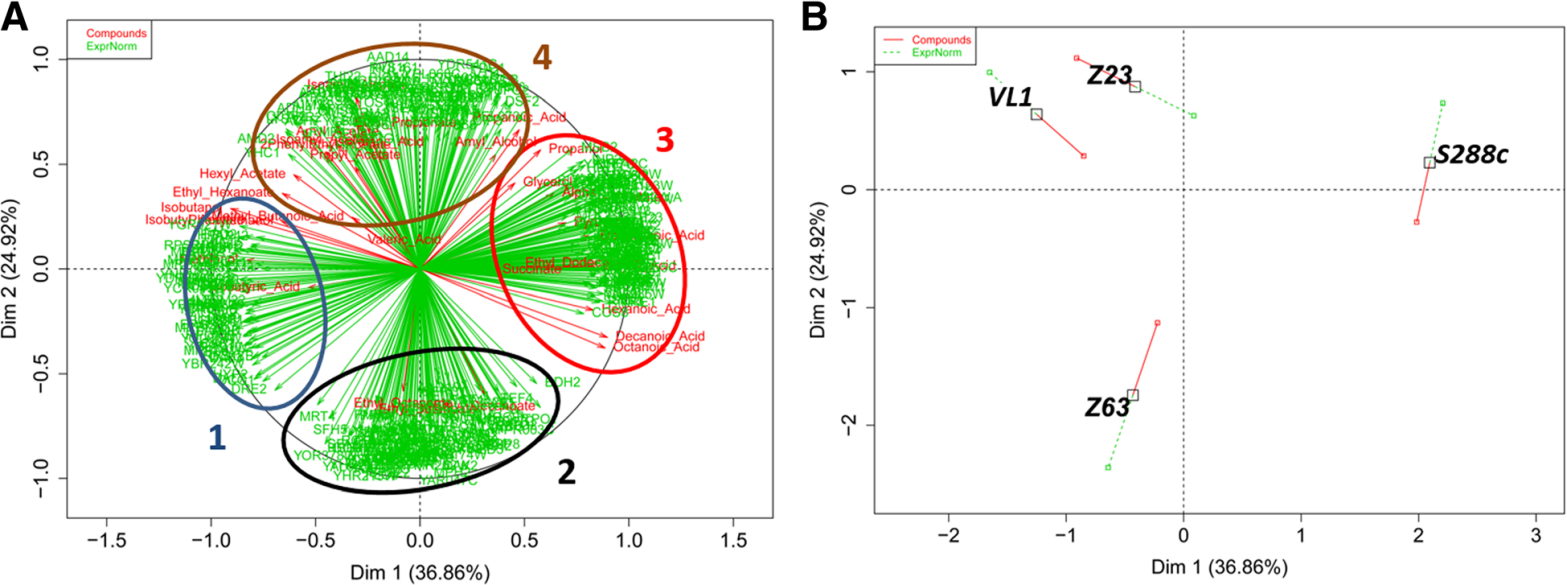
Multi-factorial analysis of GC-MS, HPLC and transcriptomic data for the four strains tested, at T1 (5 g/L). Circles 1–4 indicates groups of genes and compounds sharing similar results regarding their positioning in the image: **a** – distribution of the quantified compounds (*red*) and genes (*green*). **b** – distribution of the four tested strains

**Fig. 4:**
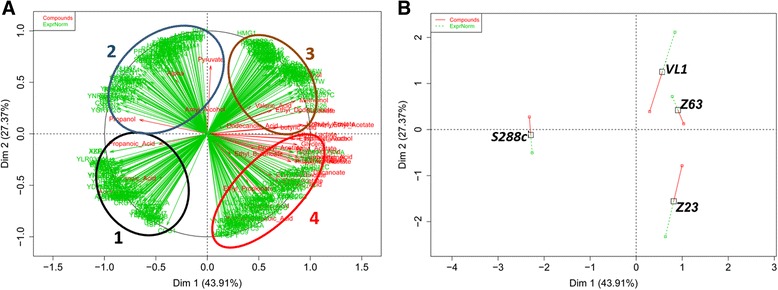
Multi-factorial analysis of GC-MS, HPLC and transcriptomic data for the four strains tested, at T2 (50 g/L). Circles 1–4 indicates groups of genes and compounds sharing similar results regarding their positioning in the image: **a** – distribution of the quantified compounds (*red*) and genes (*green*). **b** – distribution of the four tested strains

Aiming to unravel new associations between genes and aromatic compounds production we next performed a combined analysis of transcriptomic and metabolic data sets. A supervised exploratory approach sPLS-DA was carried out from gene expression data in order to select the 400 most differential expressed genes (200 for each axis) at each time point (from the 6200 *S. cerevisiae* probes present in the microarray). At the two time points, multiple factorial analysis (MFA) was then performed from expression levels of the 400 chosen genes and the 38 metabolic variables (Figs. 3 and 4). The 400 genes clustered into four main groups together with metabolites, allowing a clear separation of the strains on the basis of their gene expression and metabolic profiles. GeneCodis [54, 66, 67] was used to determine biological annotations with statistical relevance associated with the genes present in each group (Additional files 2 and 3: Tables S2 and S3).

During the growth phase (T1, Fig. 3), the reference strain S288c differed from the other yeasts (sake, cachaça and wine strains) by a higher expression level of genes of group 3 associated with an important production of propanol, glycerol and medium chain fatty acid, and conversely, a lower expression of genes of group 1, connected with a limited formation of isobutanol, methionol, isobutylacetate and phenylethanol. Genes of group 1 were identified as coding for ribosomal proteins (*RPL14B, RPS24A, RPS25B, RPL30, RPS26B, MRPL23, RPS17B, RPL40B* and *RPL26A*), involved in the structural integrity of ribosome. The association of genes coding for ribosomal proteins, with the differential production of higher alcohols and the ester isobutyl acetate (Additional file 2: Table S2), could suggest an impact of higher growth rates on the production of these compounds. It is well known that the formation of higher alcohols depends of the reduction from the respective aldehyde with the oxidation of NADH into NAD^+^ [68]. Consequently, the need for rapid production of oxidised NAD^+^ could have an important regulatory role in the formation of these compounds, explaining their higher formation by cachaça, wine and sake strains compared with the laboratory yeast. Regarding group 3, it contains genes associated with MAPK signalling pathway, cysteine and methionine metabolism and ABC transporters. The presence in this group of *ATM1*, coding for a mitochondrial exporter of Fe-S clusters and of genes from metabolism of cysteine, usually the limiting component in glutathione synthesis, suggests a more important response of S288c to oxidative stress compared with the other yeasts, generating a limitation of reductive power in this strain. This decrease may be the driving factor of the formation of several volatile fatty acids such as octanoic acid, decanoic acid, hexanoic acid, butyric acid and dodecanoic acid, which was increased in the laboratory strain. It is also tempting to speculate that *PDR5* may be involved in the export of the fatty acids. MFA also revealed that cachaça yeast (Z63) differentiated from the other strains by an increased production of ethyl esters, namely ethylbutanoate, ethyldecanoate and ethyloctanoate while VL1 and Z23 exhibited higher capacities of production of hexylacetate, propylacetate, 2-phenylethylacetate, amylalcohol, isovaleric acid, isoamylacetate, amylacetate, ethilpropionate, propanoic acid and isoamylalcohol (Additional file 2: Table S2). Interestingly, genes that were more expressed specifically in Z63 are related with metabolism of butanoate, tyrosine, beta-alanine and fatty acids, and also associated with glycolysis and gluconeogenesis. Thus, the overexpression of genes involved in the butanoate and more general in fatty acid metabolism, may directly explain the increased production of ethylbutanoate and of the other ethyl esters. Finally, no relevant biological annotation was found among the genes overexpressed in wine and sake yeast (group 4), pointing to a role of each of the genes individually.

At T2 (Fig. 4), a clear separation was also observed between strain S288c and the other strains, being this related with overexpression of genes from groups 1 and 2 versus downregulation of those of group 3 and 4 in the lab strain. In addition, S288c is characterised by an important formation of unpleasant or neutral compounds, in particular acids that contribute with unpleasant odors to wine. Genes from group 1, such as *TDH3, FBP26, SLT2, MIG2* and *GDH1,* which clustered with acids formation, were associated with central carbon metabolism and its regulation, cation transport and cell wall. Thus, the maintenance of ionic homeostasis in the interaction with the environment may appear as a determining factor in the production of the unpleasant acids. Consequently, the manipulation of specific cation homeostasis and cell wall integrity pathway could be a way of avoiding/reducing their production. Genes from group 2 included once again the term “ribosomes” but associated with the formation of alpha-ketoglutarate and pyruvate in addition to the production of higher alcohols (propanol, amylalcohol), as evidenced at T1. The other biological annotations associated with group 2 genes included purine or pyrimidine metabolism, and no clear scenario could be established between gene functions and the compounds produced. Genes from groups 3 and 4 were clearly related with the central carbon metabolism and formation of aroma compounds and are associated with marked increased concentrations of higher alcohols and ethyl and acetate esters for the fermentative yeasts, including several acetate and ethyl esters that contribute to the “floral” and “fruity” characteristics of wine (Additional file 3: Table S3). Specifically, VL1 and Z63 strains were characterised by an overexpression of genes from group 3 combined with a downregulation of those of group 2. Group 3 included a set of 17 genes related with biosynthesis of secondary metabolites, which clearly related with the production of the metabolic compounds, being more specifically associated with the terms “steroid biosynthesis”, “propanoate metabolism” (*ALD6*, *ACS2* and *ERG10*), “valine, leucine, isoleucine and lysine degradation” (*ALD6*, *ERG10*, *ERG13*), and “fatty acid metabolism” (*FAA1*, *ALD6* and *ERG10*). This could be associated to an increase production of valeric acid but also succinate, methionol and isobutanol. Group 4 genes, which differentiated strain Z23 from the others, were mainly associated with the production of a high variety of acetate and ethyl ethers. Functional categories more significantly associated with this group of genes were c-compound metabolism and oxidation-reduction process.

## Conclusions

In this work we performed the transcriptomic and metabolic characterization of four *S. cerevisiae* strains, with different origins and technological applications and unravelled new associations between genes and aromatic compounds production. Results showed differences between cachaça, sake and wine strains metabolism and gene expression, significant differences being found mainly between cachaça and sake strains, in comparison with the wine strain. However, although each strain comes from a different industrial application, we must caution that it may not be a standard representative of that industry, as strain differences are often found for the same industrial application [69]. At T1 of fermentation, strain Z63 (cachaça) showed major differences from sake and wine strains, mainly regarding the production of the ethyl esters, ethyl decanoate and ethyl octanoate. These differences were associated with the expression of genes related with the metabolism of butanoate, tyrosine, beta-alanine and fatty acids. At T2, a different scenario was found in which the sake strain (Z23) had the most distinctive behaviour when considering both metabolites produced and transcription results. At this point this strain showed a higher production of several acetate and ethyl esters and an increase in the expression of genes of c-compound metabolism and oxidation-reduction process. On the contrary, wine and cachaça strains showed an upregulation of genes related with steroid biosynthesis, propanoate metabolism, valine, leucine, isoleucine and lysine degradation, and fatty acid metabolism.

In summary, the integration of several technologies (HPLC, GC-MS, microarrays) applied to fermentation results of four strains with diverse origins and technological applications, analysed using several data analysis methods (PCA, MFA) revealed successful to understand and clarify the genes and the pathways that lead to the formation of metabolic compounds that contribute to the wine aroma and flavour. The results also show that the use of Z23 strain in a wine fermentation will produce a major amount of ethyl acetate which contributes to the fruity and floral characteristics of wine. The knowledge here obtained has the potential to be deeply explored and extended to other strains and other metabolic pathways, within an approach using aroma production as the primary selection criteria. The majority of the genes identified in this work as having their expression changed in correlation with the aroma compounds produced, play a central role in the metabolism of *S. cerevisiae*, namely *ADH6, ADH7*, *AAD6*, *ALD2*, *ALD6*, *FAA1*, *ACS2, ERG10* and *ERG13*. These genes are potential targets for gene deletion/overexpression programs using these and/or other strains, in order to better understand their role and their correlation with the aroma production network of *S. cerevisiae.* Moreover, the information now obtained may be useful in breeding programs to drive the selection of yeast strains with improved aromatic properties.

## Additional files

Additional file 1: Table S1. Concentration (mg/L) of aromatic compounds determined by GC-MS and HPLC for the four *Saccharomyces cerevisiae* strains and at two time points. (DOCX 26 kb)

Additional File 2: Table S2. List of genes present in each group of Fig. 3, together with their function, obtained after GeneCodis analysis regarding biological annotations with statistical relevance at T1. (XLSX 9 kb)

Additional File 3: Table S3. List of genes present in each group of Fig. 4, together with their function, obtained after GeneCodis analysis regarding biological annotations with statistical relevance at T2. (XLSX 16 kb)

## Abbreviations

CE: Capillary electrophoresis
CoA: Coenzyme A
DEPC: Diethylpyrocarbonate
DIMS: Direct injection mass spectrometry
FDR: False discovery rate
GC: Gas chromatography
GEO: Gene expression omnibus
GO: Gene ontology
HPLC: High performance liquid chromatography
LC: Liquid chromatography
MAPK: Mitogen-activated protein kinase
MCFA: Medium-chain fatty acid
MFA: Multivariate factorial analysis
Min: Minutes
MS: Mass spectroscopy
MS300: Synthetic must
NAD^+^: Nicotinamide adenine dinucleotide
NADH: Nicotinamide adenine dinucleotide (reduced form)
NMR: Nuclear magnetic resonance
PCA: Principal componente analysis
RNA: Ribonucleic acid
Rpm: Revolutions per minute
sPLS-DA: Sparse partial least square – discriminant analysis
YPD: Yeast extract-peptone-dextrose
