# Supplementary material for: Integrating transcriptomics and metabolomics for the analysis of the aroma profiles of Saccharomyces cerevisiae strains from diverse origins
Source: BMC Genomics. 2017 Jun 8;18:455. doi: 10.1186/s12864-017-3816-1 (PMC5465573; doi:10.1186/s12864-017-3816-1)
Supplement: Supplementary file 1 — Concentration (mg/L) of aromatic compounds determined by GC-MS and HPLC for the four Saccharomyces cerevisiae strains and at two time points. (DOCX 26 kb) [file 12864_2017_3816_MOESM1_ESM.docx]

**Table 1**: Concentration (mg/L) of aromatic compounds determined by GC-MS and HPLC for the four *Saccharomyces cerevisiae* strains and at two time points.

| Strain | Z63 | | S288c | | Z23 | | VL1 | |
| --- | --- | --- | --- | --- | --- | --- | --- | --- |
| Time points | T1 | T2 | T1 | T2 | T1 | T2 | T1 | T2 |
| Ethyl acetate | n.d. | 13.54 ± 2.79 | n.d. | 7.044 ± 4.738 | n.d. | 17.42 ± 2.96 | n.d. | 10.26 ± 3.43 |
| Ethyl propionate | 0.082 ± 0.011 | 0.255 ± 0.096 | n.d. | 0.182 ± 0.075 | 0.111 ± 0.015 | 0.267 ± 0.045 | 0.109 ± 0.024 | 0.147 ± 0.053 |
| Propyl acetate | 0.013 ± 0.001 | 0.130 ± 0.034 | 0.012 ± 0.001 | 0.076 ± 0.039 | 0.019 ± 0.004 | 0.100 ± 0.017 | 0.014 ± 0.003 | 0.071 ± 0.011 |
| 2-methylpropyl acetate | 0.016 ± 0.0002 | 0.194 ± 0.060 | n.d. | 0.032 ± 0.018 | 0.024 ± 0.004 | 0.150 ± 0.030 | 0.019 ± 0.003 | 0.147 ± 0.023 |
| Ethyl butanoate | 0.007 ± 0.001 | 0.202 ± 0.059 | 0.003 ± 0.004 | 0.134 ± 0.059 | n.d. | 0.144 ± 0.015 | 0.002 ± 0.004 | 0.087 ± 0.013 |
| Propanol | 4.21 ± 0.021 | 14.22 ± 2.17 | 7.89 ± 1.25 | 17.119 ± 1.845 | 6.125 ± 0.491 | 7.05 ± 0.45 | 6.225 ± 0.438 | 7.57 ± 0.24 |
| Ethyl 2-methylbutanoate | n.d. | n.d. | n.d. | n.d. | n.d. | n.d. | n.d. | n.d. |
| Ethyl 3-methylbutanoate | n.d. | n.d. | n.d. | n.d. | n.d. | n.d. | n.d. | n.d. |
| 2-methylpropanol | 5.00 ± 0.020 | 1.873 ± 0.438 | 2.79 ± 0.58 | 10.214 ± 0.478 | 5.817 ± 0.233 | 17.17 ± 1.87 | 6.388 ± 0.273 | 28.22 ± 1.38 |
| 2-methylbutylacetate | 0.001 ± 0.0002 | 28.17 ± 5.03 | n.d. | 0.005 ± 0.001 | 0.002 ± 0.0002 | 0.010 ± 0.002 | 0.0012 ± 0.0004 | 0.0078 ± 0.0015 |
| 3-methylbutylacetate | 0.064 ± 0.006 | 0.011 ± 0.003 | 0.04 ± 0.01 | 0.756 ± 0.272 | 0.195 ± 0.019 | 2.047 ± 0.337 | 0.108 ± 0.034 | 1.473 ± 0.270 |
| Ethyl valerate | n.d. | 1.873 ± 0.438 | n.d. | 0.024 ± 0.011 | n.d. | 0.074 ± 0.012 | n.d. | 0.053 ± 0.010 |
| Isoamyl alcohol | 24.19 ± 1.37 | 141.2 ± 20.3 | 31.13 ± 5.98 | 92.872 ± 2.905 | 46.28 ± 2.44 | 132.49 ± 10.43 | 38.766 ± 0.504 | 125.59 ± 14.48 |
| Ethyl hexanoate | 0.063 ± 0.019 | 0.756 ± 0.160 | 0.040 ± 0.001 | 0.439 ± 0.159 | 0.083 ± 0.005 | 0.775 ± 0.049 | 0.073 ± 0.011 | 0.474 ± 0.112 |
| 2-methylbutanol | 0.035 ± 0.005 | 0.029 ± 0.003 | 0.049 ± 0.009 | 0.024 ± 0.001 | 0.043 ± 0.007 | 0.020 ± 0.002 | 0.044 ± 0.004 | 0.0195 ± 0.0021 |
| Hexyl acetate | 0.113 ± 0.006 | 0.288 ± 0.034 | 0.025 ± 0.035 | 0.111 ± 0.040 | 0.273 ± 0.037 | 0.258 ± 0.019 | 0.171 ± 0.046 | 0.167 ± 0.021 |
| Ethyl lactate | n.d. | 0.690 ± 0.177 | n.d. | 0.308 ± 0.032 | n.d. | 0.549 ± 0.051 | n.d. | 0.513 ± 0.103 |
| Ethyl octanoate | 0.169 ± 0.078 | 1.508 ± 0.323 | 0.069 ± 0.007 | 0.728 ± 0.139 | 0.096 ± 0.019 | 1.439 ± 0.046 | 0.069 ± 0.028 | 1.238 ± 0.261 |
| Propanoic acid | 0.084 ± 0.015 | 0.211 ± 0.043 | 0.198 ± 0.047 | 0.231 ± 0.034 | 0.160 ± 0.014 | 0.167 ± 0.015 | 0.153 ± 0.015 | 0.144 ± 0.014 |
| Isobutyric acid | 0.20 ± 0.007 | 0.295 ± 0.035 | 0.116 ± 0.003 | 0.124 ± 0.028 | 0.200 ± 0.037 | 0.344 ± 0.027 | 0.155 ± 0.016 | 0.288 ± 0.043 |
| Butyric acid | 0.184 ± 0.016 | 0.598 ± 0.045 | 0.252 ± 0.004 | 0.476 ± 0.102 | 0.176 ± 0.023 | 0.565 ± 0.031 | 0.180 ± 0.013 | 0.584 ± 0.058 |
| Ethyl decanoate | 0.04 ± 0.01 | 0.644 ± 0.127 | 0.033 ± 0.003 | 0.373 ± 0.023 | 0.033 ± 0.004 | 0.810 ± 0.099 | 0.019 ± 0.003 | 0.618 ± 0.104 |
| 3-methylbutanoic acid | 0.09 ± 0.0001 | 0.219 ± 0.023 | 0.095 ± 0.002 | 0.188 ± 0.038 | 0.139 ± 0.028 | 0.350 ± 0.032 | 0.101 ± 0.009 | 0.255 ± 0.056 |
| 2-methylbutanoic acid | 0.06 ± 0.002 | 0.134 ± 0.015 | 0.050 ± 0.002 | 0.115 ± 0.025 | 0.078 ± 0.018 | 0.179 ± 0.022 | 0.057 ± 0.005 | 0.122 ± 0.030 |
| Methionol | 0.07 ± 0.002 | 0.295 ± 0.060 | 0.032 ± 0.005 | 0.035 ± 0.003 | 0.070 ± 0.011 | 0.157 ± 0.020 | 0.068 ± 0.011 | 0.226 ± 0.053 |
| Valeric acid | 0.002 ± 0.003 | 0.007 ± 0.002 | 0.002 ± 0.002 | 0.003 ± 0.002 | 0.002 ± 0.0004 | 0.004 ± 0.002 | 0.003 ± 0.004 | 0.005 ± 0.001 |
| 2-phenylethyl acetate | 0.02 ± 0.023 | 0.282 ± 0.032 | 0.009 ± 0.001 | 0.063 ± 0.010 | 0.088 ± 0.020 | 0.320 ± 0.017 | 0.043 ± 0.014 | 0.347 ± 0.037 |
| Hexanoic acid | 0.74 ± 0.110 | 4.133 ± 0.304 | 0.976 ± 0.003 | 2.500 ± 0.526 | 0.650 ± 0.148 | 3.99 ± 0.52 | 0.570 ± 0.032 | 3.55 ± 0.60 |
| Ethyl dodecanoate | 0.01 ± 0.001 | 0.097 ± 0.040 | 0.012 ± 0.003 | 0.048 ± 0.012 | 0.014 ± 0.002 | 0.138 ± 0.029 | 0.003 ± 0.0002 | 0.209 ± 0.051 |
| 2-phenylethanol | 1.59 ± 0.029 | 5.535 ± 0.866 | 0.954 ± 0.057 | 2.312 ± 0.307 | 1.988 ± 0.220 | 7.95 ± 0.74 | 1.524 ± 0.084 | 6.71 ± 0.66 |
| Octanoic acid | 0.54 ± 0.031 | 3.671 ± 0.422 | 0.941 ± 0.021 | 2.333 ± 0.452 | 0.254 ± 0.053 | 3.73 ± 0.55 | 0.100 ± 0.028 | 2.58 ± 0.46 |
| Decanoic acid | 0.039 ± 0.001 | 0.169 ± 0.055 | 0.083 ± 0.017 | 0.221 ± 0.053 | 0.017 ± 0.003 | 0.168 ± 0.047 | 0.006 ± 0.002 | 0.092 ± 0.029 |
| Dodecanoic acid | n.d. | 0.031 ± 0.021 | 0.025 ± 0.012 | 0.028 ± 0.011 | 0.009 ± 0.002 | 0.029 ± 0.008 | 0.001 ± 0.002 | 0.030 ± 0.007 |
| Glycerol | 1718.37 ± 401.81 | 5319.69 66.95 | 2067.32 ± 54.02 | 3889.33 ± 88.99 | 2097.71 ± 28.53 | 4979.84 ± 206.59 | 1772.08 ± 13.29 | 4534.07 ± 80.17 |
| Succinic acid | 362.37 ± 88.14 | 665.36 ± 16.34 | 366.96 ± 6.49 | 383.79 ± 7.94 | 357.82 ± 3.88 | 558.63 ± 36.22 | 327.17 ± 3.62 | 578.86 ± 8.58 |
| Acetic acid | 83.60 ± 18.43 | 88.38 ± 2.08 | 217.46 ± 5.13 | 284.42 ± 0.99 | 134.80 ± 5.93 | 197.28 ± 15.84 | 91.51 ± 3.31 | 71.99 ± 10.31 |
| Pyruvic acid | 69.64 ± 16.74 | 65.72 ± 0.19 | 87.51 ± 1.61 | n.d. | 73.25 ± 2.50 | 53.42 ± 3.19 | 68.93 ± 1.01 | n.d. |
| Alpha-ketoglutaric acid | 117.66 ± 41.75 | 125.74 ± 1.18 | 172.23 ± 2.90 | 121.34 ± 3.43 | 143.99 ± 1.93 | 102.58 ± 5.79 | 132.37 ± 1.23 | 112.75 ± 3.78 |
